# Ferric carboxymaltose vs. ferrous sulfate for the treatment of anemia in advanced chronic kidney disease: an observational retrospective study and cost analysis

**DOI:** 10.1038/s41598-021-86769-z

**Published:** 2021-04-02

**Authors:** Luigi Cirillo, Chiara Somma, Marco Allinovi, Alfredo Bagalà, Giuseppe Ferro, Elio Di Marcantonio, Stefania Bellelli, Lorenzo Antonio Dallari, Piercarlo Ballo, Pietro Claudio Dattolo

**Affiliations:** 1 Nephrology and Dialysis Unit, Santa Maria Annunziata Hospital, Bagno a Ripoli, Florence, Italy; 2 Nephrology, Dialysis and Transplant Unit, Careggi University Hospital, Florence, Italy; 3 Health Technology Assessment and Management (HTAM) Research Area, IRES Piemonte, Torino, Italy; 4 Cardiology Unit, Santa Maria Annunziata Hospital, Bagno a Ripoli, Florence, Italy

**Keywords:** Chronic kidney disease, Anaemia

## Abstract

In non-dialysis-dependent chronic kidney disease (NDD-CKD), erythropoiesis-stimulating agents (ESAs) and iron supplementation are essential for anemia management. Ferric carboxymaltose (FCM) is a relatively novel intravenous iron formulation used in different clinical settings, although scarce data exist in NDD-CKD patients. Primary objective of this study was to retrospectively evaluate the efficacy of FCM compared with oral ferrous sulfate for the treatment of iron-deficiency anemia in a cohort of NDD-CKD patients, considering also the treatment costs. This was a monocentric, retrospective observational study reviewing 349 NDD-CKD patients attending an outpatient clinic between June 2013 and December 2016. Patients were treated by either FCM intravenous infusion or oral ferrous sulfate. We collected serum values of hemoglobin, ferritin and transferrin saturation (TSAT) and ESAs doses at 12 and 18 months. The costs related to both treatments were also analysed. 239 patients were treated with FCM intravenous infusion and 110 patients with oral ferrous sulfate. The two groups were not statistically different for age, BMI and eGFR values. At 18 months, hemoglobin, serum ferritin and TSAT values increased significantly from baseline in the FCM group, compared with the ferrous sulfate group. ESAs dose and rate of infusion decreased only in the FCM group. At 18 months, the treatment costs, analysed per week, was higher in the ferrous sulfate group, compared with the FCM group, and this was mostly due to a reduction in ESAs prescription in the FCM group. Routine intravenous FCM treatment in an outpatient clinic of NDD-CKD patients results in better correction of iron-deficiency anemia when compared to ferrous sulfate. In addition to this, treating NDD-CKD patients with FCM leads to a significant reduction of the treatment costs by reducing ESAs use.

## Introduction

Anemia is a common condition in patients with chronic kidney disease (CKD) and it is associated with increased morbidity and mortality^1,2^. Global anemia prevalence in 2010 was estimated at 32.9%, causing 68.36 million years lived with disability with an increasing contribution from CKD^3^.

The prevalence and severity of anemia are directly related to the degree of renal failure^4^, and in this specific pathological condition it is caused by a multifactorial etiopathogenetic mechanism: decreased erythropoietin production by failing kidneys, iron deficiency due to dietary restriction, decreased red blood cell half-live, decreased erythropoietic response in the bone marrow related to inflammation and uremic toxins, increased hepcidin levels related to chronic inflammation, which also contributes to anemia by downregulating both intestinal iron absorption and release of stored iron, and determining hypo responsiveness to erythropoiesis-stimulating agents (ESAs)^1,5^. Due to this heterogeneous etiopathogenesis, a substantial number of patients remains anemic, despite currently available therapies^4^.

The Kidney Disease Improving Global Outcomes (KDIGO) guidelines, in order to correct iron deficiency, recommend to start iron therapy before ESAs therapy^5,6^. Iron replacement includes the use of oral or intravenous iron formulations^6,7^. Oral iron formulations are often poorly absorbed, and not well tolerated because of adverse gastrointestinal events^8^. To overcome these side effects, intravenous iron preparations were developed, and their use is especially indicated when iron absorption is compromised or a rapid replacement is required^9,10^.

The intravenous iron preparations include different formulations with different drug administration frequency and different adverse events profile^8^. Older high-molecular weight intravenous iron dextrans have been associated with hypersensitivity reactions limiting their use^8,11–13^. Ferric carboxymaltose (FCM) is an intravenous iron formulation which does not contain dextran and interacts with the reticuloendothelial system with reduced release of free iron, allowing controlled iron delivery into target tissues^9,10^. Despite recent studies increasing our knowledge on iron treatment for dialysis patients, such as the PIVOTAL study^14^, in non-dialysis-dependent CKD (NDD-CKD) patients, the selection of oral versus intravenous administration is less definite; the choice should take into account the severity of anaemia, availability of venous access, response to prior therapy, patient adherence and costs^5,7^, particularly for those in advanced CKD stages. Moreover, long-term follow-up studies in this population are scarce^6,7^, and only one trial, the FIND-CKD study, has been performed so far^14^.

The primary objective of our study was to evaluate the efficacy of intravenous FCM compared with oral ferrous sulfate for the treatment of iron-deficiency anemia in a cohort of outpatients with advanced NDD-CKD. The primary efficacy outcome was the mean change of hemoglobin from baseline to the highest observed value after 12 and 18 months of treatment. Secondary efficacy measures included the mean changes from baseline to the highest observed ferritin and transferrin saturation (TSAT) values at 12 and 18 months.

Moreover, to provide an economic evaluation of the treatments, the direct medical cost of intravenous FCM compared with oral ferrous sulfate treatment have been estimated.

## Methods

### Study design

In this monocentric, retrospective, observational study, all adult outpatients affected by advanced CKD attending the Nephrology outpatient clinic have been consecutively included from June 2013 to December 2016. Their medical records were reviewed and followed retrospectively for up to 18 months since the time of inclusion. Sociodemographic and clinical data were collected by electronic medical records of the outpatient clinic, with patient names replaced by alphanumeric codes for anonymization. The study protocol was approved by SMA Hospital Institutional Review Board requirements and the Declaration of Helsinki. All methods were performed in accordance with required guidelines and regulations. All patients included in the study signed informed consent.

### Patient population

The patients included in our study were older than 18 years of age, affected by advanced NDD-CKD with an estimated glomerular filtration rate (eGFR) ≤ 20 ml/min/1.73 m^2^ (measured by CKD-EPI equation) and had iron-deficiency anemia requiring iron supplementation for at least 90 days.

As defined by the 2012 KDIGO guidelines^6^, the diagnosis of iron-deficiency anemia was based on two serum hemoglobin concentrations ≤ 11.0 g/dl within 7 days, together with ferritin ≤ 500 ng/ml and TSAT ≤ 30%, and a stable ESAs therapy.

**Figure 1:**
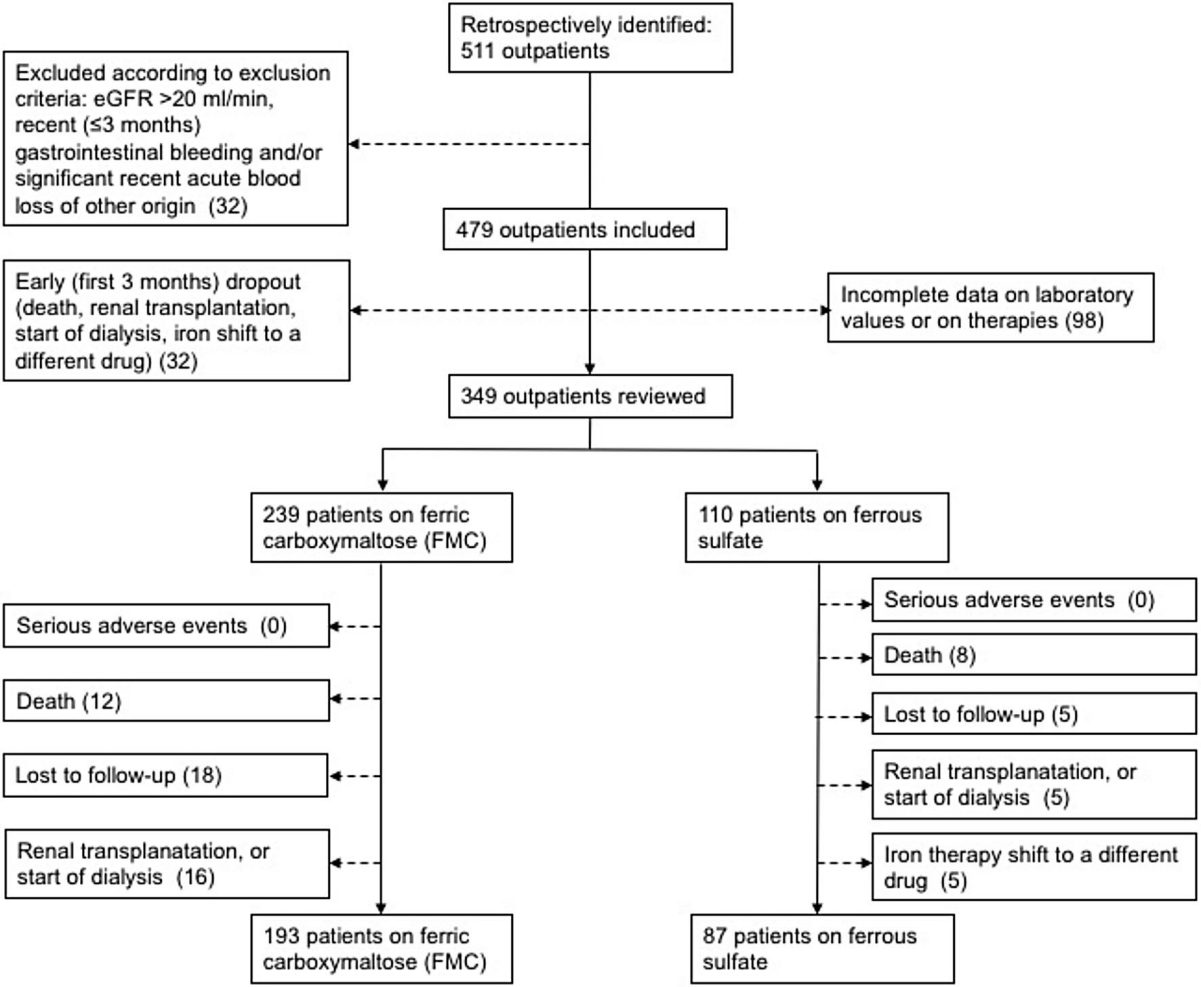
Study design.

Exclusion criteria included eGFR > 20 ml/min/1.73 m^2^, recent (≤ 3 months) gastrointestinal bleeding and/or significant recent acute blood loss of other origin, incomplete medical records or incomplete follow-up. Patients were also excluded from final analyses in case of early (< 3 months) iron therapy shift to a different drug, renal transplantation, start of dialysis, death during the follow-up. Study design and patients sample are presented in Fig. 1.

### Treatment

Eligible patients were divided into two groups depending on the iron therapy at the time of enrollment. Subjects on FCM therapy (group 1) received a single dose of 10–15 mg/kg (up to a maximum of 1000 mg), as recommended in product leaflet, in a slow intravenous infusion over 20 min. Subjects on ferrous sulfate (group 2) received a dose of 325 mg (105 mg of elemental iron) given orally one or two times daily. The dose of iron supplementation required was decided with a target-oriented approach aimed to achieve and maintain ferritin ≥ 500 ng/ml and TSAT ≥ 30%. Following the first infusion of FMC or ferrous sulfate prescription, dose adjustments were evaluated at intervals of about 2–4 weeks. None of the patients in group 1 received oral iron therapy and none in group 2 received intravenous iron treatments during the follow-up. The ESAs used in our study were epoietin α or darbepoetin; dose adjustment was decided targeting hemoglobin level between 10 and 12 g/dl and according to each drug pharmacokinetics.

Serious adverse events, considering reactions having death, life-threatening, or hospitalization need as outcome, were recorded; blood pressure at FCM infusion was reported also.

### Cost analysis

The treatment costs for both the FMC and the ferrous sulfate group, stratified according to administration of epoietin α or darbepoetin, were calculated and analyzed.

Direct medical costs referred to “a standard” pharmacological treatment. The drugs costs were taken from the ESTAR registry (Regional Technical Administrative Support Authority, Italian NHS) and ESTAR’s prices are the NHS purchase price. The costs evaluation has been made with the regional health system perspective and data have been expressed in Euros (2018). At baseline, cost per patient per week (inclusive of iron use and ESAs costs) was € 32.7 for FCM and € 32.2 for ferrous sulfate. The cost of the first 12 months of pharmacological treatment administered to patients in group 1 or in group 2 was calculated by multiplying the cost of treatment per patient per week, by the number of weeks in a year (52.17) and by the number of patients treated at 12 months follow-up, stratified by administration of epoietin α and darbepoetin.

The cost of the first 18 months of pharmacological treatment administered to the patients in group 1 and in group 2 was calculated by adding the treatment cost of the first 12 months to that of the subsequent 6 months.

### Statistical analysis

Data were expressed as number (proportion) for categorical variables and mean (± SD, standard deviation) for normally distributed continuous data and median (IQR, interquartile range) for non-normally distributed continuous data. Differences in the distribution of patient characteristics at the baseline were assessed using the Fisher’s exact test or the Pearson’s Chi-squared test for categorical variables and the two-sided two-sample t test for normal continuous variables or two-sample Wilcoxon rank-sum (Mann–Whitney) test for non-parametric continuous variables.

The comparisons between the two study groups were performed using the Student’s t test for independent samples, whereas within-group differences were explored using the Student’s t test for paired data. Three-way ANOVA was used to explore differences in the cost of therapy between groups over time, considering iron therapy (FMC vs ferrous sulfate), ESAs (epoietin α vs darbepoetin), and time (baseline, 12 and 18-month follow-up) as main factors.

Drug costs have been expressed as mean (± SD). The significance level was set at 0.05. All tests were 2-tailed. All analyses were performed using SPSS (Statistical Package for Social Science) for Windows, Release 22.0 (SPSS Inc., Chicago, IL).

## Results

From 2013 to 2016, a total of 511 outpatients were diagnosed with advanced NDD-CKD; 162 did not fulfill the eligibility criteria. The exclusion criteria were applied and a total of 349 patients were analyzed into two treatment groups: FMC (group 1, n = 239, 68.5%), and ferrous sulfate (group 2, n = 110, 31.5%). Excluded patients, according to single criteria at the different time points, are presented in Fig. 1.

### Baseline characteristics

Baseline demographic, clinical and laboratory characteristics are shown in Table 1. There were no statistically significant differences in any considered variable between the two groups at baseline. In particular, the two groups were homogeneous by age (respectively 70.7 ± 14.0 vs 73.7 ± 13.8 years; p = 0.6), BMI (respectively 26.1 ± 4.2 vs 23.6 ± 4.7 kg/m^2^; p = 0.4), serum hemoglobin levels (respectively 9.7 ± 1.5 g/dl vs 9.8 ± 1.6 g/dl; p = 0.4), serum ferritin levels (respectively 103 ± 91 ng/ml vs 102 ± 112 ng/ml; p = 0.7), TSAT (respectively 19.4 ± 9% vs 19.8 ± 8%; p = 0.8) and eGFR (respectively 12.0 ± 4.1 vs 12.7 ± 3.9; p = 0.7).

**Table 1:** Baseline characteristics of patient population.

Characteristics		Study groups	
	Total (n = 349)	Group 1 FCM (n = 239)	Group 2 Ferrous sulfate (n = 110)
Age (mean ± SD, years)	71.2 ± 13.8	70.7 ± 14.0	73.7 ± 13.8
BMI (mean ± SD, kg/m^2^)	26.1 ± 4.6	26.1 ± 4.2	26.3 ± 4.7
Body weight (mean ± SD, kg)	75.9 ± 15.9	75.7 ± 15.6	76.1 ± 16
CRP (mean ± SD, mg/dl)	1.0 ± 1.2	1.0 ± 1.2	0.9 ± 1.3
ESAs use [n, (%)]	194 (56%)	132 (55%)	62 (56%)
Hb (mean ± SD, g/dl)	9.7 ± 1.6	9.7 ± 1.5	9.8 ± 1.6
TSAT (mean ± SD, %)	19.5 ± 9	19.4 ± 9	19.8 ± 8
Ferritin (mean ± SD, ng/ml)	103 ± 91	103 ± 83	102 ± 112
eGFR (mean ± SD, ml/min/1.73mq)	12.2 ± 4.53	12.0 ± 4.1	12.7 ± 3.9
sCr (mean ± SD, mg/dl)	4.2 ± 1.8	4.1 ± 1.7	4.2 ± 1.9

### Treatment

During the 18-month treatment phase, the mean cumulative dose of iron received was 1.73 ± 0.65 g in group 1 and 84.6 ± 21.5 g in group 2. The majority of participants in group 1 (124 patients, 52%) were administered an average of 1500 mg of iron, while 43 patients (18%) received 1000 mg, and 72 patients (30%) received 2000 mg. A total of 132/239 participants (55%) in group 1 and 62/100 subjects (56%) in group 2 were treated with ESAs at baseline, with similar prescribed doses of epoietin α and darbepoetin.

### Treatment outcomes

At 12 months, response to treatment was observed in both groups (Table 2). In group 1, hemoglobin significantly increased from baseline after 12 months (9.7 ± 1.5 vs 11.9 ± 1.8 g/dl, p < 0.05), similarly serum ferritin (103 ± 83 vs 275 ± 151 ng/ml, p < 0.05) and TSAT (19.4 ± 9 vs 29.9 ± 12, p < 0.05); in group 2 hemoglobin (9.8 ± 1.6 vs 10.1 ± 1.8 g/dl, p = 0.4), serum ferritin (102 ± 112 vs 180 ± 132 ng/ml, p = 0.1) and TSAT (19.8 ± 8 vs 22.0 ± 11, p = 0.1) increased without reaching significance.

**Table 2:** Iron blood tests and hemoglobin at different time points in the two study groups.

	Transferrin saturation (%)	Ferritin (ng/ml)	Hemoglobin (mg/dl)	Erythropoietin therapy n (%)
**Group 1 (FCM therapy)**				
Baseline (n = 239)	19.4 ± 9	103 ± 83	9.7 ± 1.5	132 (55%)
12-months follow-up (n = 212)	29.9 ± 12	275 ± 151	11.9 ± 1.8	110 (52%)
	p < 0.05	p < 0.05	p < 0.05	p = 0.8
Baseline (n = 239)	19.4 ± 9	103 ± 83	9.7 ± 1.5	132 (55%)
18-months follow-up (n = 193)	31.0 ± 17	310 ± 193	11.9 ± 1.9	89 (46%)
	p < 0.05	p < 0.01	p < 0.05	p = 0.3
**Group 2 (ferrous sulfate therapy)**				
Baseline (n = 110)	19.8 ± 8	102 ± 112	9.8 ± 1.6	62 (56%)
12-months follow-up (n = 96)	22.0 ± 11	180 ± 132	10.1 ± 1.8	60 (62%)
	p = 0.1	p = 0.1	p = 0.4	p = 0.6
Baseline (n = 110)	19.8 ± 8	102 ± 112	9.8 ± 1.6	62 (56%)
18-months follow-up (n = 87)	24.0 ± 12	175 ± 145	9.9 ± 1.7	56 (64%)
	p = 0.4	p = 0.1	p = 0.7	p = 0.4

At 18 months, in patients from group 1 hemoglobin (9.7 ± 1.5 vs. 11.9 ± 1.9 g/dl, p < 0.05), serum ferritin (103 ± 83 vs 310 ± 193 ng/ml, p < 0.01) and TSAT (19.4 ± 9 vs 31.0 ± 17, p < 0.05) still improved. Conversely, in patients from group 2 no significant further increase in hemoglobin (9.8 ± 1.6 vs. 9.9 ± 1.7 g/dl, p = 0.7), serum ferritin (102 ± 112 vs 175 ± 145 ng/ml, p = 0.1) and TSAT (19.8 ± 8 vs 24.0 ± 12, p = 0.4) was recorded and the concentrations remained comparable to the values reached at 12 months (Table 2).

In addition to this, ESAs utilization decreased in group 1, considering the number of treated patients and prescribed doses: at 18 months, comparing the two groups, group 1 showed a significant reduction of treated patients vs group 2 (respectively 89/193 vs 56/87 subjects, p < 0.01), epoietin α dose (3500 ± 1563 vs 5672 ± 2145, p < 0.05) and darbepoetin dose (14.9 ± 4.95 vs 26.4 ± 11.0, p < 0.05) (Table 3).

**Table 3:** Epoietin α and darbepoetin therapy at different time points in the two study groups.

	Baseline	12 months	18 months
**Epoietin α (IU/week)**			
FCM	4940 ± 1985	4120 ± 1782	3500 ± 1563
Ferrous sulfate	4850 ± 2010	5356 ± 2312	5672 ± 2145
**Darbepoetin (IU/week)**			
FCM	23.2 ± 8.4	15.2 ± 5.9	14.9 ± 4.95
Ferrous sulfate	22.9 ± 9.3	25.2 ± 9.9	26.4 ± 11.0

### Adverse reactions

No patient showed serious adverse reactions to the treatment. Blood pressure did not show a significantly change before and after the FCM infusion (systolic 127 ± 12 mmHg vs 130 ± 13 mmHg and diastolic 77 ± 9 mmHg vs 75 ± 9 mmHg; p = 0.619 and p = 0.679, respectively). Similarly, on subsequent visits, blood pressure did not differ from baseline values.

### Cost analysis

**Figure 2:**
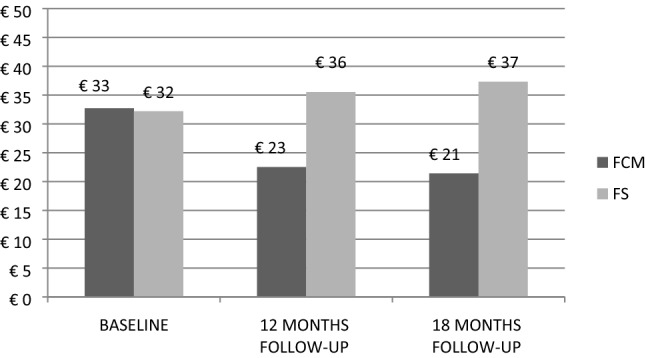
Cost of therapy (including ESAs cost) per person/week for patients treated with FCM and ferrous sulfate (FS) by time of treatment.

Therapy costs per person per week for group 1, including patients on epoietin α and darbepoetin, decreased from € 32.70 at baseline, to € 22.50 at 12 months (− 31.2%) and to € 21.40 at 18 months (− 34.6% vs baseline). Therapy costs per person per week for group 2, including patients on epoietin α and darbepoetin, increased from € 32.20 at baseline, to € 35.50 at 12 months (+ 10.2%) and to € 37.30 at 18 months (+ 15.8%). Therapy costs per person per week for group 1 and group 2 have been represented by time of treatment in Fig. 2.

Cost analysis is summarized in the Supplementary Data (Table 1). After 18 months, the cost of therapy per patient per week decreased in group 1 and increased in group 2 (p < 0.001 by three-way ANOVA).

In group 1, an overall cost decrease of € 43.384 between 12 months and baseline has been estimated for each one of 110 patients (of whom 22 were treated with epoietin α and 88 with darbepoetin) and an overall cost reduction of € 36.086 between 18 months and baseline has been estimated for each one of 89 patients (of whom 18 were treated with epoietin α and 71 with darbepoetin).

In group 2, an overall cost growth of € 7.137 between 12 months and baseline has been estimated for each one of 60 patients (of whom 12 were treated with epoietin α and 48 with darbepoetin) and an overall cost increase of € 8.441 between 18 months and baseline has been estimated for each one of 56 patients (of whom 11 were treated with epoietin α and 45 with darbepoetin). It is possible that the annual saving following FCM treatment and the annual cost increase with ferrous sulfate could have been even higher, due to the lack of data of patients that died during the 18-month period.

## Discussion

In this study, we found that intravenous FCM treatment is cost-saving and effective compared to oral ferrous sulfate in NDD-CKD patients, considering also ESAs use. Importantly, we found a positive response despite the advanced CKD stage of our cohort, which is the main strength of the population included in our study, possibly indicating that FCM is an effective therapy also in very advanced NDD-CKD patients. These considerations are in line with previous data showing a better laboratory and clinical outcome in NDD-CKD patients treated with intravenous iron preparations^5^, although previous clinical trials mostly recruited patients with a higher mean eGFR. In a randomized clinical trial, Qunibi et al. showed that FCM is more effective and better tolerated than oral ferrous sulfate in a cohort of 250 NDD-CKD patients, mostly with CKD stages 3b and 4^15^. In the FIND-CKD study, MacDougall et al. recruited 626 NDD-CKD patients with demographic and clinical characteristics similar to our population, except for a higher eGFR (32.2 vs. 12.3 ml/min/1.73 m^2^) and a lower ferritin level at baseline (57.1 vs. 103 µg/l)^14^. FCM was more effective than oral ferrous sulfate in reaching and maintaining Hb level, and delaying the use of ESAs^14^. Compared with their results, at 12 months the increase of Hb levels in our population was higher (2.2 vs 1.4 g/dl), probably due to higher basal ferritin levels and concurrent use of ESAs of our cohort. Similar data in NDD-CKD patients emerged in the REPAIR-IDA trial^16,17^, where FCM was shown to be a safe and effective alternative to intravenous iron sucrose, even if this latter requires generally multiple injections of higher concentration to guarantee adequate iron level. The increase of Hb reported was lower than the present study at the end of follow-up and this may be due to the longer observation period of our study.

Also, our results seem to confirm data from other larger comparative studies including dialysis patients^18^, and NDD-CKD patients^19–22^, as well as patients with different pathological conditions, such as gastrointestinal bleeding^23^, cancer^24^ and CHF^22^. Despite other previous reports from clinical trials^14,15^, oral iron use was not accompanied by significant Hb increase in our group at the end of the follow-up. We speculate this observation may be due to three reasons. First, the extremely advanced stage of NDD-CKD (mean eGFR 12 ml/min/1.73 m^2^) of our cohort, which makes our cohort similar to dialysis-dependent patients, where oral iron is generally less effective^6,8^; second, due to the nature of the study, differently from published trials^14,15^, we cannot express certainty about the real compliance to the therapy. This latter point is supported by the small difference in Hb values between the oral iron group and the FCM group in the FIND-CKD study, where patient compliance in the oral iron group was systematically assessed^14^. Our results however seem to confirm data from “real-world” clinical practice where, despite also the use of ESAs, several studies suggest that the management of anemia in advanced CKD and at dialysis initiation is suboptimal: in this setting, about 30–50% of patients have Hb < 10 g/dl^25,26^. Lastly, another factor potentially explaining the different response could be the dose of iron administered to the two groups; this was not compared due to the retrospective nature of the study.

We also performed a cost analysis that showed a reduction in the cost for FCM therapy compared to ferrous sulfate therapy. FCM use was associated with reduced ESAs dose and decreased total number of patients needing ESAs at the end of the follow-up, both important determinants for reducing overall expenditure and balancing the higher cost of FCM compared with ferrous sulfate. Other direct medical costs (e.g. minor adverse reaction treatment) should also be taken into account, although they have not been analyzed in this study. However, as previously reported by Dahl et al*.*, who compared another intravenous iron drug (Ferumoxytol) with oral iron, the majority of the overall cost of the intravenous iron monotherapy was attributed to drug acquisition costs, while the total cost of oral iron monotherapy was mostly composed of costs associated with adverse events^27^. Considering that efficacy and safety for intravenous iron formulations (FCM and Ferumoxytol) are comparable^9^, including costs of adverse reaction treatment in our cost analysis may have potentially made FCM even more cost-effective. Moreover, reducing the ESAs use may lead to a reduction of the risk of vascular access thrombosis, cardiovascular events and tumors, which have been associated with high ESAs doses^13^. Data on the economic impact of FCM therapy in an outpatient clinic are consistent with results from few other cost-analyses on intravenous iron use in NDD-CKD patients. Comparing different intravenous iron-containing drugs, two studies showed a cumulative reduction of direct (e.g. drug, staff, injection materials) and indirect (e.g. travel to hospital, day-off from work) costs varying from 19 to 68% using FCM, rather than iron sucrose in an outpatient setting^28,29^, while they did not analyze the concurrent use of ESAs as we did in our study. To this regard, Toblli et al*.* showed that switching to FCM therapy gives significant improvement in hematological and iron parameters and a significant reduction in ESAs dose requirements in patients with oral iron-refractory anemia^30^. In particular, they reported mean ESAs consumption was significantly reduced by 80%, similarly to our population where the decrease was 65%. In another Italian pilot study, Minutolo et al.^31^ also found a significant reduction of prescribed ESAs dose, generating a cost-saving per patient of € 637 in 24 weeks of observation despite the higher cost of FCM. Moreover, similar findings from economic perspective come from other settings: Calvet et al. in a study of 282 patients affected by iron deficiency anemia and been treated before surgery for colorectal cancer, found a cost saving of € 274 per patient, again suggesting the advantage of using FCM therapy instead of oral iron^32^.

As in previously published papers^9,10,18,20^, we did not report hypersensitivity reactions (anaphylactic type) or other serious adverse events. Due to the retrospective nature of the study, we could not systematically register all the minor side events of the iron medications.

## Strength and limitations

Our study has some limitations. First, due to its observational nature, we could have not considered important influential factors, such as the compliance to oral iron therapy and the previous treatment, that may reduce the impact of cost-effectiveness if a significant number of patients were on oral iron before the switch to intravenous iron; moreover, the total iron dosages of FCM infusion and oral ferrous sulfate per group have been not presented. Second, this study was conducted at a single-center outpatient clinic, which may result in possible selection-bias, prescription-bias and observer-bias. Third, we did not include some direct medical costs (specialist visits, staff costs and administration costs), direct non-medical costs (injections materials, transfusions and patient transport, caregiver assistance) and indirect costs (productivity loss of working days). Lastly, the conclusions of the study should be applied in particular to late-stage CKD patients, according to the characteristics of the cohort.

However, the main strengths of our study are the cohort size, which is the largest population with late-stage CKD studied so far, and the follow-up period for patients treated with FCM therapy, which is the longest reported so far in the literature, to our knowledge. These elements should be taken into account in routine clinical practice when correcting advanced anemia of extreme stages of NDD-CKD patients, who can be comparable to dialysis patients, where intravenous iron medications should be preferred.

## Conclusion

In conclusion, this analysis suggests that FCM routinely used in an outpatient clinic of NDD-CKD patients is associated with a significantly better correction of iron-deficiency anemia when compared to ferrous sulfate. Our data, which present the longest follow-up in NDD-CKD patients on intravenous FCM therapy, showed that FCM therapy led to anemia correction, maintaining this steady result for at least 18 months, and reduced the prescribed ESAs dose in patients with advanced CKD. This advantage is accompanied by economic benefits derived from the overall reduction costs mainly due to reduction of ESAs utilization. According to these results, we can speculate that FCM represents a valid therapeutic and economical option also in more advanced CKD stages.

## Supplementary Information

Supplementary Table 1.
