# Supplementary material for: Ferric carboxymaltose vs. ferrous sulfate for the treatment of anemia in advanced chronic kidney disease: an observational retrospective study and cost analysis
Source: Sci Rep. 2021 Apr 2;11:7463. doi: 10.1038/s41598-021-86769-z (PMC8018957; doi:10.1038/s41598-021-86769-z)
Supplement: Supplementary file 1 — Supplementary Table 1. [file 41598_2021_86769_MOESM1_ESM.docx]

**Supplementary Table 1.** Cost of therapy in patients treated with FCM iron versus those treated with ferrous sulfate.

From “Ferric carboxymaltose vs. ferrous sulfate for the treatment of anemia in advanced chronic kidney disease: an observational retrospective study and cost analysis”.

L Cirillo, C Somma, M Allinovi, A Bagalà, G Ferro, ED Marcantonio, S Bellelli, L Dallari, P Ballo, PC. Dattolo.

| **Group of patients** |  | **Baseline**  **(N. patients; Cost per patient/week)** | **12 months (N. patients; Cost per patient/week)** | **18 months**  **(N. patients; Cost per patient/week)** | **Cost difference from 18 months FUP to baseline** | |
| --- | --- | --- | --- | --- | --- | --- |
|  |  |  |  |  | **per patient/week** | **per patient/year** |
| **Patients on Erythropoietin** | | | | | | |
| **FCM** | N. Patients | 26 | 22 | 18 | - | - |
|  | Cost per patient | € 6 | € 5 | € 4.3 | -€ 1.7 | -€ 88.7 |
| **Ferrous sulfate** | N. Patients | 12 | 12 | 11 | - | - |
|  | Cost per patient | € 5.9 | € 6.5 | € 6.9 | +€ 1 | +€ 52.17 |
| **Patients on Darbepoetin** | | | | | | |
| **FCM** | N. Patients | 106 | 88 | 71 | - | - |
|  | Cost per patient | € 26.7 | € 17.5 | € 17.1 | -€ 9.6 | -€ 500.8 |
| **Ferrous sulfate** | N. Patients | 50 | 48 | 45 | - | - |
|  | Cost per patient | € 26.3 | € 29.0 | € 30.4 | +€ 4.1 | +€ 213.9 |
